# Routes to Diagnosis for Suspected Sarcoma: The Impact of Symptoms and Clinical Findings on the Diagnostic Process

**DOI:** 10.1155/2016/8639272

**Published:** 2016-12-26

**Authors:** Heidi Buvarp Dyrop, Peter Vedsted, Mathias Rædkjær, Akmal Safwat, Johnny Keller

**Affiliations:** ^1^Sarcoma Center of Aarhus University Hospital, Aarhus C, Denmark; ^2^Department of Experimental Clinical Oncology, Aarhus University Hospital, Aarhus C, Denmark; ^3^The Research Unit for General Practice, Research Center for Cancer Diagnosis, Aarhus University, Aarhus C, Denmark; ^4^Department of Oncology, Sarcoma Center of Aarhus University Hospital, Aarhus C, Denmark; ^5^Department of Orthopedics, Sarcoma Center of Aarhus University Hospital, Aarhus C, Denmark

## Abstract

*Background and Objectives*. Sarcoma patients often experience delay before diagnosis. We examined the association between presenting symptoms/signs and time intervals for suspected sarcoma patients.* Methods*. 545 consecutive patients suspected for sarcoma referred over a one-year period were included. Median time intervals in routes to diagnosis were collected from medical records and questionnaires.* Results*. 102 patients (18.7%) had a sarcoma; 68 (12.5%) had other malignancies. Median interval for the patient (time from first symptom to first doctor visit), primary care, local hospital, sarcoma center, diagnostic, and total interval for sarcoma patients were 77, 17, 29, 17, 65, and 176 days, respectively. Sarcoma patients visited more hospital departments and had longer median primary care (+10 days) and diagnostic intervals (+19 days) than patients with benign conditions. Median primary care (−19 days) and sarcoma center (−4 days) intervals were shorter for patients with a lump versus no lump. Median patient (+40 days), primary care (+12 days), diagnostic (+17 days), and total intervals (+78 days) were longer for patients presenting with pain versus no pain. GP suspicion of malignancy shortened local hospital (−20 days) and total intervals (−104 days).* Conclusions*. The main part of delay could be attributed to the patient and local hospitals. Length of time intervals was associated with presenting symptoms/signs and GP suspicion.

## 1. Introduction

Sarcoma is one of the rarer cancer types and patients are often prone to delay before diagnosis [1, 2]. Whether this affects prognosis is debated. Some studies show that long symptom duration improves survival [3, 4]; others show a poorer survival with increasing symptom duration [5, 6], and some show no difference [7–11]. For other cancer types, a review concluded that an expedited diagnosis improved cancer outcomes overall, but that this varied with cancer type [12]. Further, lower use of the English 2-Week Wait pathway by GPs has been associated with an increased mortality among cancer patients [13], but no specific results for sarcomas are presented in these two studies. Apart from affecting prognosis, delays may affect patients' evaluations and give rise to psychological distress and patient complaints [14, 15].

Fast track referral pathways have been implemented in some countries to reduce delays [16–18], and the Danish Cancer Patient Pathways (CPPs) have reduced time between referral to a specialized sarcoma center and initiation of treatment in sarcoma patients [19]. However, this is only a small part of the pathway as the main part of the diagnostic route lies with the patient, the general practitioner (GP), and local hospitals. Approximately 85% of all cancer patients in Denmark initiate their diagnostic route in general practice [20], and GPs are important in sarcoma diagnosis. This task is not easy as only one in 100 soft tissue lumps are malignant [21] and with a yearly incidence of 330 sarcomas among 3,400 GPs (in average one sarcoma every 10 year per GP) a GP very seldom in a career will diagnose a sarcoma [22]. Furthermore, the CPPs are based on alarm symptoms qualifying the patient for referral to the fast track-pathway as follows:

Description of the alarm symptoms qualifying the patient for referral to the Danish Sarcoma Cancer Patient Pathway (CPP) is as follows:

- Soft tissue tumor over five cm.
- Soft tissue tumor situated on or below the deep muscle fascia.
- Rapidly growing soft tissue tumor.
- Palpable bone tumor.
- Deep, persisting bone pains without other obvious explanation.

Patients without alarm symptoms may thus experience delays.

Studies have investigated presenting symptoms among confirmed sarcoma patients at time of diagnosis in highly specialized sarcoma centers, and the symptom duration is usually reported as a total sum from first symptom to diagnosis. However, this approach sheds no light on the initial symptoms and does not include the population of benign tumors from which sarcomas have to be separated. Thus, we need detailed information on the milestones and how the presenting symptoms affect the length of time intervals to be able to optimize the diagnostic pathway for sarcoma patients.

We aimed to examine time intervals, symptom presentation, and routes to diagnosis from first perceived symptom to diagnosis at a specialist center among patients referred to the CPP for sarcomas. We hypothesized that the time to diagnosis for suspected sarcoma patients differs depending on the presenting signs and symptoms.

## 2. Materials and Methods

### 2.1. Setting

The study was performed at Aarhus Sarcoma Center (ASC), one of the two centralized sarcoma centers in Denmark, with a catchment area of approximately 2.5 million inhabitants. ASC functions mainly as the highly specialized sarcoma department, to which all patients found to have a suspicion of sarcoma at local hospitals are referred. Further, ASC also serves as the local orthopedic hospital department for suspected sarcoma patients living in Aarhus Municipality (approximately 330.000 inhabitants).

### 2.2. Study Population and Data Collection

All consecutive patients referred to the CPP for sarcoma at ASC in the period from 1st of September 2014 to 31st of August 2015 were invited to participate. Data were collected from patient questionnaires and medical records. A patient and a GP questionnaire was developed based on similar questionnaires for other cancer types [24] and adapted for sarcoma patients. Both questionnaires were pilot tested to ensure understanding before startup of data collection.

Patients received their questionnaire by mail before the first appointment at ASC and were encouraged to answer questions beforehand. Patients were interviewed after the appointment, to ensure correct completion of questions. An informed consent was also provided at this time. The GP questionnaire was sent to the patients' GP if either the medical record showed or the patient stated that they had visited their GP in relation to the present pathway. GPs received no remuneration. GPs were reminded with a new questionnaire after 4-5 weeks, followed by a telephone reminder after a further three weeks. The patient's route to diagnosis was tracked backwards and data from local hospitals were collected from medical records. Final diagnosis and treatment were collected from medical records containing pathology reports at ASC.

### 2.3. Variables

Tumor grade for sarcoma patients was classified by the Trojani classification system [23]. For analyses, grades 2 and 3 were defined as high grade and grade 1 and locally aggressive/rarely metastasizing tumors as low grade tumors.

Tumor size was measured as the largest diameter on MRI or CT. If none of these scans were performed, size was taken from the pathology and, if not removed, from ultrasound, x-ray, or clinical measurement. For analyses on time intervals in different tumor size groups, only patients where the size had been measured on an MRI/CT or histology report were included. Tumor depth was classified as subcutaneous or subfascial relative to the deep muscle fascia.

Questions about primary symptoms and development in symptoms were answered by the patients in free text, and each reported symptom was coded with an individual number. No grouping of symptoms into categories was done during the recording. The recorded codes could then later be collected into larger groups suitable for analyses.

The GPs were asked to report their tentative/suspected diagnosis in free text. Each diagnosis was coded with individual numbers using the same approach as for presenting symptoms, and all codes corresponding to a suspicion of any malignancy were classified as GP suspicion being present.

**Figure 1:**
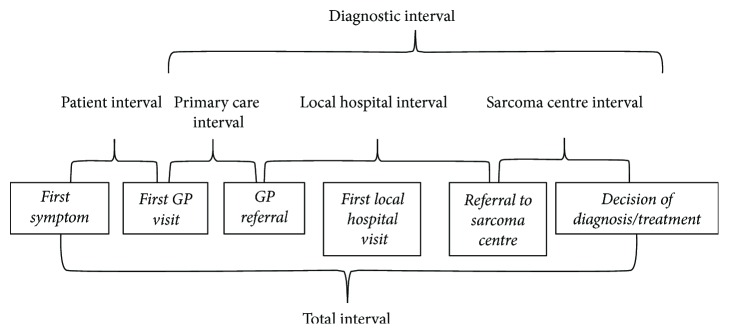
Overview of time points and calculated time intervals [23].

Patients reported date of symptom debut and date of first doctor visit. GPs reported date of first visit and date of referral for further investigation at hospitals. Date of first appointment and date of referral for each local hospital department were collected from medical records. From ASC the date of received referral and date of decision of diagnosis and/or initial treatment were collected. If only a month and year were stated in questionnaires, the 15th of that month was chosen as the specific date. If only a year was stated, the 1st of July in that year was chosen as the specific date. For patients with missing GP data, the patient reported date for first doctor visit was used to calculate patient interval and diagnostic interval. Time intervals are measured in calendar days and defined in accordance with the Aarhus Statement [25]. We calculated six time intervals; patient, primary care, local hospital, sarcoma center, diagnostic, and total interval (Figure 1). Patient interval was defined as time from first symptom to first doctor visit, primary care interval as time from the first GP visit to GP referral to hospital, local hospital interval as time from referral to first local hospital to final referral to the sarcoma center, and the sarcoma center interval as time from received referral to the date where a decision on the final course of treatment was made (decision of a final treatment modality or decision of no treatment). This decision date was also the end point of the diagnostic and total interval. It was chosen as end point to ensure comparativeness of time intervals between patients regardless of final diagnosis. The treatment interval is thus not included. The starting points of the diagnostic interval and the total interval were the first doctor visit and the date of first symptom, respectively.

### 2.4. Ethical Approval

The study was approved by the Danish Data Protection Agency (journal number 2007-58-0010). All patients provided written consent to participation. Approval from the Committee on Health Research Ethics of the Central Denmark Region was not needed according to Danish law.

### 2.5. Statistical Analysis

Descriptive statistics were used to test differences between participants and nonparticipants (chi-squared test (gender) and Wilcoxon Rank Sum Test (age)). Number of hospital departments visited and number of GP consultations were compared with the Wilcoxon Rank Sum test. Time intervals are reported as medians with interquartile intervals (IQI). Comparisons of time intervals at the 50th and 75th percentile between different groups were performed with quantile regression analyses, using the procedure written by Miranda [26]. Gender distribution was found to be equal in all groups and was thus not adjusted for. Age differed between groups and was adjusted for as a categorical variable (<20, 20–39, 40–59 and ≥60 years). Quantile regression analyses were repeated with adjustments for both age and gender to assess the effect of gender. This resulted in no or very small changes in estimated differences, thus supporting our decision to exclude the gender variable in our reported analyses. *p* values of 5% or less were considered significant, and all *p* values are two-sided. Statistical calculations were performed using Stata® statistical software, version 13.

## 3. Results

### 3.1. Patient and GP Participation

During the inclusion period a total of 607 patients entered the sarcoma CPP at ASC. Of these, 545 patients were included as 56 patients did not want to participate in the study, five were not mentally able to answer questionnaires, and one did not speak Danish or English. Nonparticipants did not differ significantly from participants with regard to age or gender. 466 GP questionnaires were sent out, of which 400 were completed. For 42 patients with a nonresponding GP, information on dates and performed imaging investigations at the GPs office could be collected from the GP referral or the medical records.

### 3.2. Patient and Tumor Characteristics

Of 545 included patients, 102 were diagnosed with a sarcoma and 68 with other malignancies, giving a total proportion of malignancies of 31.2%. There were no significant differences in gender (*p* = 0.911) between sarcoma patients, patients with other malignancies, and patients with benign conditions (Table 1). There was a significant difference in age distribution among these three groups (*p* = 0.0001, Kruskal-Wallis test), and patients with other malignancies had the highest median age (Table 1). There were 56 patients below the age of 18, of which eight were diagnosed with a sarcoma and eight with other malignancies. The most frequent sarcomas were liposarcoma (*n* = 20), malignant fibrous histiocytoma/undifferentiated pleomorphic sarcoma (*n* = 12), and leiomyosarcoma (*n* = 9). The most frequent other malignant diagnoses were metastasis (*n* = 30), lymphoma (*n* = 23), and myelomatosis (*n* = 6). Most frequent benign diagnoses were lipoma (*n* = 60), reactive tissue changes (*n* = 46), and schwannoma/neurofibroma (*n* = 23). Forty-five sarcomas were grade 3 tumors, 24 were of grade 2, 25 were of grade 1, and eight were locally aggressive/rarely metastasizing tumors. Seven sarcoma patients had metastases at time of diagnosis. Further patient and tumor characteristics are summarized in Table 1.

### 3.3. Routes to Diagnosis

Most frequent reasons for seeking medical care for the total patient population were pain, wanting to know what it was, consulting for something else, being urged by others, and incidental findings on imaging (Table 2). 59.2% of patients with benign conditions and 55.9% of sarcoma patients had pain related to their tumor, and there was no statistically significant difference in the proportion of patients with pain in the two groups, *p* = 0.547. A complete list of all presenting symptoms can be found in Table 3. Further characteristics of the patients' routes to diagnosis are presented in Table 4. The majority had first presented to their GP (83.7%). The number of local hospital departments visited between the GP and ASC was statistically significantly higher both for sarcoma patients compared to patients with benign conditions (*p* = 0.001) and for patients with other malignancies compared to patients with benign conditions (*p* < 0.001). There was a trend towards a higher number of GP consultations for sarcoma patients compared to patients with benign conditions (*p* = 0.051).

### 3.4. Time Intervals

Median numbers of calendar days with interquartile intervals (IQI) for all time intervals are presented in Table 5. Overall, the longest intervals were seen for the patient interval and the local hospital interval contributing to a median total interval of 155 days where 25% that waited longest had a time interval of 423 days from first symptom to decision. In general, differences in symptoms and signs modified some of the intervals. Note especially that presence of pain prolonged the intervals and GP suspicion shortened the intervals.

Table 6 presents the estimated differences in time intervals at the 50th and 75th percentile level adjusted for age. Patients with sarcoma tended to have longer time intervals compared to patients with benign conditions. For patients with other malignancies the reverse relationship was found, as these patients had shorter time intervals than patients with benign conditions. The median sarcoma center interval was approximately one week statistically significantly longer for patients with other malignancies compared to patients with benign conditions. Sarcoma patients with high grade tumors had a significantly shorter median total interval compared to sarcoma patients with low grade tumors due to a shorter patient interval, whereas the diagnostic interval was longer for high grade tumors.

It is worth noticing that patients presenting with a lump tended to have a longer patient interval compared to patients without a lump, whereas the primary care interval and sarcoma center intervals were statistically significantly shortened (−19 days and −4 days, resp.). For patients presenting at the sarcoma center with a tumor over 5 cm, the patient interval and thus the total interval were statistically significantly longer compared to patients with smaller tumors (+24 days and +43 days, resp.). Patients with subfascial soft tissue tumors had a statistically significantly shorter patient interval (−31 days) compared with patients with subcutaneous tumors.

Focusing on 25% of patients waiting longest (the 75th percentile) accentuated the described differences.

## 4. Discussion

### 4.1. Summary of Main Results

The GP was involved in the diagnostic route for the majority of patients, and the main reason for help seeking was pain. Patient interval and local hospital interval constituted the main parts of the total time from first symptom to diagnosis. Sarcoma patients had longer time intervals and patients with other malignancies had shorter time intervals compared to patients with benign conditions. Patients with malignancies visited more local hospital departments than patients with benign conditions. Presence of a lump, large tumor size, and presence of pain increased patient intervals, whereas patients with subfascial tumor location and high malignancy grade had shorter patient interval. High tumor grade and presence of pain increased health system intervals, whereas large tumor size, presence of a lump, and initial GP suspicion shortened health system intervals. Differences were more pronounced at the 75th percentile level.

### 4.2. Strengths and Limitations

The strengths of our study lie in a high participation rate and high completeness of data. Nonparticipants were similar in age and gender distribution to participating patients, but we have no information on the number of malignancies or the length of time intervals among nonparticipants. However, the small number of nonparticipants limits the effect of this possible selection bias. Regarding nonparticipating GPs, it could be that GPs of patients with delays would decline to answer, which would cause an underestimation of time intervals. To minimize this problem, we used the patient reported dates to calculate the patient interval and diagnostic interval for patients with missing GP response. Nonetheless, the primary care interval may be underestimated. Patient reported data were validated with interviews, improving the completeness and data quality. GPs were encouraged to consult medical records to reduce recall bias.

### 4.3. Comparison with Literature

We confirmed that time intervals differed depending on presenting symptoms. The presence of pain increased time intervals, possibly due to the fact that in general practice pain is a common symptom with low positive predictive value for serious disease. Pain was also the main reason for sarcoma patients to seek help, but the use of pain as an alarm symptom in sarcomas is debated, and it has been suggested to remove this feature from referral guidelines [27, 28]. In the Danish sarcoma CPP, pain is defined as an alarm symptom only for bone sarcoma, and 12 out of 14 bone sarcomas in our study had pain. However, around 50% of the soft tissue sarcomas presented with pain, thus contradicting the perception that soft tissue sarcomas are not painful. Unfortunately, around 50% of patients with benign tumors had pain as well, making this feature a poor independent discriminator of malignancy, and further research on the use of pain as an alarm symptom is thus needed.

Deeply situated tumors had shorter time intervals in our study, which has also been previously reported [29]. Regarding tumor size, other authors report that tumors larger than 5 cm have shorter time intervals [29], and this was also seen for the local hospital interval in our study. Further, our patients presenting with a lump had a significantly shorter primary care interval, which supports conclusions from other studies that presence of alarm symptoms results in shorter time intervals [30–32]. However, the patient interval was longer for patients with a lump and patients with tumors over 5 cm in our study, contradicting the findings by George and Grimer of a shorter patient interval for larger tumors [29]. Our finding could be a result of tumor growth during prolonged waiting time, but the observational design of our study limits the ability to conclude causality.

The proportion of patients with an initial GP suspicion of malignancy was about one-third for sarcomas, suggesting that two-thirds of all sarcomas are found on vague, common, or nonspecific symptoms. This is consistent with English findings showing that sarcomas are more likely to go unnoticed in primary care and be referred outside of the Two-Week Wait referral pathway [33]. Initial GP suspicion significantly reduced the time intervals in our cohort, and this importance of GP suspicion in Denmark has been shown for other cancer types as well [34–36]. The correct selection of patients for inclusion in fast track referral programs is important as waiting times outside the fast track program may be longer to accommodate for the fast track referrals [37]. Our results indicate that the selection of patients could be suboptimal, and this needs to be addressed.

Sarcoma patients with a higher malignancy grade had shorter total time intervals in our study. This difference was mainly driven by a shorter patient interval, indicating that aggressive tumors could have more pronounced symptoms that make the patient seek help faster. It may also be a result of recall bias as patients with clearer symptoms may remember the symptom onset more precisely. More surprisingly, the diagnostic interval was longer for patients with high grade tumors. This finding contradicts results from studies on other cancer types where the patients referred under urgent referral guidelines with the shortest diagnostic intervals had a higher malignancy grade [38], probably due to confounding by indication. In our results it seems that the patients with the most aggressive tumors are not more likely to be selected for a fast track referral route, indicating that referral criteria may not be optimal as discussed earlier. Another possible reason for the longer diagnostic delay could be that longer waiting time in the later stages of the natural disease history results in a higher malignancy grade. The observational design of our study prohibits any conclusions regarding this possible reverse causation, but the matter should be investigated.

The main part of total interval was caused by the patient, followed by the local hospital interval. Other studies have attributed delay in sarcoma patients to GPs in primary care [1, 2] with GP delays ranging from 2 to 12 months, but in our material this was not the case as median primary care interval only constituted eight days. This is more similar to the primary care interval of seven days reported for both Danish lung cancer patients and English sarcoma patients [34, 39]. The GPs are thus not to blame for long delays, and the focus should be set on time spent at local hospitals. The secondary care interval has also been reported as the major contributor to delay for other cancer types with median times ranging from 11 to 21 days [40], which is still shorter compared to our median interval of 29 days for sarcoma patients. Overall, the median total interval is long for the patients in our population (155 days) compared to that of other cancer types (ranging from median 30 to 60 days) [41], and as the time intervals are highly right skewed many of our patients wait considerably longer than 155 days. There seems to be a possibility to reduce overall delay by reducing local hospital time, and this could be done by decreasing waiting time for investigations at local hospitals, for example, by providing yes/no investigations to GPs [42].

A relatively large proportion of the patients referred to the CPP had cancer. This can be explained by the selection process, with investigations at local hospitals before referral to the sarcoma center. This highlights the importance of easy and direct access to investigations from general practice. However, the selection may also be due to a wait-and-see strategy which could lead to later stage at treatment, as discussed earlier.

## 5. Conclusions

We found that time to diagnosis was associated with presenting signs and symptoms and presence of GP suspicion. Patients presenting atypically seem to experience longer waiting times before diagnosis, which may be a possible side effect of having alarm symptom based fast track referral programs such as the CPPs. The main part of the total time was caused by the patient interval and it would be relevant to look further into reducing this to support earlier diagnosis. The local hospital delay should also be addressed, for example, by providing easy and quick access to diagnostic investigations locally and optimizing referral criteria.

## Figures and Tables

**Table 1:** Patient and tumor characteristics of 545 patients referred to the Cancer Patient Pathway for sarcomas.

	Benign tumors	Other malignancies	Sarcomas
	(*n* = 375)	(*n* = 68)	(*n* = 102)

*Age* ^1^			
Median (IQI)	52.0 (36.0–64.0)	68.5 (55.5–75.0)	55.0 (44.0–70.0)
*Gender distribution* ^2^			
Female (*n* (%))	181 (48.3)	31 (45.6)	48 (47.1)
Male (*n* (%))	194 (51.7)	37 (54.4)	54 (52.9)
*Tissue type*			
Soft tissue (*n* (%))	255 (68.0)	40 (58.8)	88 (86.3)
Bone (*n* (%))	120 (32.0)	28 (41.2)	14 (13.7)
*Tumor size* ^3^			
Median (IQI)	3.2 (2.0–5.5)	3.8 (2.6–6.5)	5.75 (4.0–9.0)
Mean (SD)	4.4 (4.0)	5.4 (4.1)	7.2 (5.8)
Size over 5 cm (*n* (%))	123 (32.8)	26 (38.2)	63 (61.8)
Size under 5 cm (*n* (%))	232 (61.9)	35 (51.5)	33 (32.4)
Missing	20 (5.3)	7 (10.3)	6 (5.9)
*Geographic area*			
Aarhus Municipality (*n* (%))	117 (31.2)	11 (16.2)	15 (14.7)
Rest of Jutland area (*n* (%))	258 (68.8)	57 (83.8)	87 (85.3)

*Tumor depth for soft tissue tumors*			
	(*n* = 255)	(*n* = 40)	(*n* = 88)

Subcutaneous (*n* (%))	108 (42.4)	25 (62.5)	34 (38.6)
Subfascial (*n* (%))	147 (57.6)	15 (37.5)	54 (61.4)

^1^Age significantly differed between groups, *p* = 0.0001

^2^Gender did not differ significantly between groups, *p* = 0.911

^3^Measured on diagnostic MRI or CT for most patients. If this procedure was not performed, size was measured by pathology report if the tumour was removed. If tumor was not removed, size was measured by ultrasound if this was performed or by clinical measurement.

**Table 2:** Main reason for seeking medical care as stated by the patient.

	Benign (*n* = 375) *n* (%)	Other malignancies (*n* = 68) *n* (%)	Sarcomas (*n* = 102) *n* (%)	Total population (*n* = 545) *n* (%)
Increasing size of the tumor/swelling	20 (5.3)	3 (4.4)	7 (6.9)	30 (5.5)
Promptly reacted to the presence of swelling/lump	25 (6.7)	7 (10.3)	9 (8.8)	41 (7.5)
Tumor/swelling/pain did not disappear	24 (6.4)	3 (4.4)	6 (5.9)	33 (6.1)
Pain	77 (20.5)	17 (25.0)	15 (14.7)	109 (20.0)
Bothered to much	6 (1.6)	1 (1.5)	6 (5.9)	13 (2.4)
Afraid that it was cancer	22 (5.9)	3 (4.4)	5 (4.9)	30 (5.5)
Was worried/unsecure about the symptoms	18 (4.8)	2 (2.9)	6 (5.9)	26 (4.8)
Wanted to know what it was	34 (9.1)	1 (1.5)	12 (11.8)	47 (8.6)
Could not work/hindered at work	5 (1.3)	0 (0.0)	1 (1.0)	6 (1.1)
Restriction of movement	4 (1.1)	0 (0.0)	0 (0.0)	4 (0.7)
Hindered in daily activity	13 (3.5)	0 (0.0)	4 (3.9)	17 (3.1)
Affected night sleep	1 (0.3)	0 (0.0)	1 (1.0)	2 (0.4)
Were at the doctor's office for something else	45 (12.0)	11 (16.2)	9 (8.8)	65 (11.9)
Wanted it removed	2 (0.5)	0 (0.0)	3 (2.9)	5 (0.9)
Concerned for the cosmetic appearance	1 (0.3)	0 (0.0)	1 (1.0)	2 (0.4)
Weight loss	1 (0.3)	0 (0.0)	0 (0.0)	1 (0.2)
Thought it was side effects to medicine	1 (0.3)	0 (0.0)	0 (0.0)	1 (0.2)
Thought it was an insect bite	2 (0.5)	0 (0.0)	0 (0.0)	2 (0.4)
Urged to seek doctor by others	35 (9.3)	6 (8.8)	10 (9.8)	51 (9.4)
Wanted a referral to scanning	1 (0.3)	0 (0.0)	0 (0.0)	1 (0.2)
Had many moles and are aware of skin changes	0 (0.0)	1 (1.5)	0 (0.0)	1 (0.2)
Read cancer awareness brochure	1 (0.3)	0 (0.0)	0 (0.0)	1 (0.2)
Thought it was a hernia	0 (0.0)	1 (1.5)	0 (0.0)	1 (0.2)
Thought it was a fractured bone	0 (0.0)	1 (1.5)	0 (0.0)	1 (0.2)
Fatigue	1 (0.3)	0 (0.0)	0 (0.0)	1 (0.2)
Wanted antibiotics	1 (0.3)	0 (0.0)	0 (0.0)	1 (0.2)
Previously had cancer and are aware of any lumps	1 (0.3)	2 (2.9)	2 (2.0)	5 (0.9)
Wanted referral to physical therapy	1 (0.3)	0 (0.0)	0 (0.0)	1 (0.2)
Incidental finding on imaging	33 (8.8)	9 (13.2)	5 (4.9)	47 (8.6)

**Table 3:** Patient reported initial symptoms.

Initial symptom	Sarcomas (*n* = 102) *n* (%)	Other malignancies (*n* = 68) *n* (%)	Benign (*n* = 375) *n* (%)
Noticed lump	67 (65.7)	27 (39.7)	194 (51.7)
Noticed indentation of the skin	1 (1.0)	0 (0.0)	1 (0.3)
Mobile lump	0 (0.0)	2 (2.9)	2 (0.5)
Noticed swelling	7 (6.9)	7 (10.3)	34 (9.1)
Soft lump	1 (1.0)	1 (1.5)	2 (0.5)
Lump with discharge	0 (0.0)	0 (0.0)	1 (0.3)
Hard lump	4 (3.9)	0 (0.0)	8 (2.1)
Previously removed lump recurred	6 (5.9)	0 (0.0)	3 (0.8)
Noticed skin change/wound	4 (3.9)	3 (4.4)	3 (0.8)
Itching	1 (1.0)	0 (0.0)	4 (1.1)
Redness	1 (1.0)	0 (0.0)	5 (1.3)
Pain	27 (26.5)	24 (35.3)	135 (36.0)
Bother/pain related to pressure on lump	6 (5.9)	0 (0.0)	13 (3.5)
Night pain	1 (1.0)	0 (0.0)	5 (1.3)
Pain or stiffness in the morning	0 (0.0)	0 (0.0)	1 (0.3)
Pain related to movement	5 (4.9)	4 (5.9)	23 (6.1)
Tenderness	6 (5.9)	4 (5.9)	37 (9.9)
Radiating pain	4 (3.9)	1 (1.5)	12 (3.2)
Sensation of tightness	2 (2.0)	2 (2.9)	3 (0.8)
Reduced strength	2 (2.0)	0 (0.0)	1 (0.3)
Sensibility disturbances	3 (2.9)	1 (1.5)	8 (2.1)
Sensation of heaviness	0 (0.0)	0 (0.0)	1 (0.3)
Reduced ability to practice sports	1 (1.0)	1 (1.5)	8 (2.1)
Problems with walking	4 (3.9)	1 (1.5)	11 (2.9)
Reduction of movement ability	0 (0.0)	1 (1.5)	11 (2.9)
Started after a trauma	6 (5.9)	8 (11.8)	34 (9.1)
Started after exercise	1 (1.0)	0 (0.0)	5 (1.3)
Clicking sound from joint	0 (0.0)	0 (0.0)	2 (0.5)
Slipping sensation in joint	0 (0.0)	0 (0.0)	2 (0.5)
Joint locking	0 (0.0)	0 (0.0)	5 (1.3)
Sensation of snap in muscle	0 (0.0)	2 (2.9)	1 (0.3)
Fever	0 (0.0)	0 (0.0)	1 (0.3)
Hot flushes	0 (0.0)	0 (0.0)	2 (0.5)
Sleep disturbances	0 (0.0)	0 (0.0)	3 (0.8)
Weight loss	0 (0.0)	2 (2.9)	3 (0.8)
Lump discovered by others	5 (4.9)	1 (1.5)	19 (5.1)
Noticed blood in underwear	1 (1.0)	0 (0.0)	0 (0.0)
Bluish skin	1 (1.0)	0 (0.0)	2 (0.5)
Fatigue	0 (0.0)	2 (2.9)	8 (2.1)
Occurred in relation to pregnancy	0 (0.0)	0 (0.0)	3 (0.8)
Dizziness	0 (0.0)	1 (1.5)	2 (0.5)
Occurred after mononucleosis	0 (0.0)	0 (0.0)	1 (0.3)
Hematuria	2 (2.0)	1 (1.5)	0 (0.0)
Flank pain	2 (2.0)	0 (0.0)	0 (0.0)
Fracture	1 (1.0)	1 (1.5)	0 (0.0)
Occurred after insect bite	1 (1.0)	0 (0.0)	1 (0.3)
Yellow skin	0 (0.0)	1 (1.5)	0 (0.0)
Shortness of breath	0 (0.0)	1 (1.5)	0 (0.0)
Constipation	0 (0.0)	0 (0.0)	1 (0.3)
Night sweat	0 (0.0)	1 (1.5)	0 (0.0)
Intermittent pain	0 (0.0)	0 (0.0)	9 (2.4)
Headache	0 (0.0)	0 (0.0)	1 (0.3)
Warm skin	0 (0.0)	0 (0.0)	1 (0.3)

Patients may have more than one initial presenting symptom.

**Table 4:** Routes to diagnosis for 545 patients referred to the Cancer Patient Pathway for sarcomas.

	Benign (*n* = 375)	Other malignancies (*n* = 68)	Sarcomas (*n* = 102)	Total population (*n* = 545)
*First physician patient presented to (n (%))*				
GP	320 (85.3)	47 (69.1)	89 (87.3)	456 (83.7)
Private specialist	3 (0.8)	1 (1.5)	2 (2.0)	6 (1.1)
Hospital doctor	44 (11.7)	18 (26.5)	9 (8.8)	71 (13.0)
Out of hours GP	2 (2.9)	2 (2.9)	2 (2.0)	9 (1.7)
Other^1^	3 (0.8)	0 (0.0)	0 (0.0)	3 (0.6)
*GP initially suspected malignancy* ^2^				
Yes	94 (30.6)	25 (46.8)	27 (32.9)	146 (33.5)
No	213 (69.4)	22 (53.2)	55 (67.1)	290 (66.5)
*Number of GP visits* ^3^				
Mean (SD)	1.4 (0.9)	1.5 (1.0)	1.6 (1.1)	1.4 (0.9)
Median (IQI)	1 (1-1)	1 (1-2)	1 (1-2)	1 (1-2)
*Number of hospital departments visited* ^4^				
Mean (SD)	0.8 (0.7)	1.1 (0.6)	1.1 (0.7)	0.9 (0.7)
Median (IQI)	1 (0-1)	1 (1-1)	1 (1-1)	1 (0-1)
*Referral with histological diagnosis of sarcoma (n (%))*				
Yes	4 (1.1)	5 (7.4)	31 (30.4)	40 (7.3)
No	371 (98.9)	63 (92.7)	71 (69.6)	505 (92.7)
*Referred with regrowth of previously removed tumor (n (%))*				
Yes	4 (1.1)	1 (1.5)	11 (10.8)	16 (2.9)
No	371 (98.9)	67 (98.5)	91 (89.2)	529 (97.1)
*Referred after incidental findings on imaging (n (%))*				
Yes	33 (8.8)	9 (13.2)	5 (4.9)	47 (8.6)
No	342 (91.2)	59 (86.8)	97 (95.1)	498 (91.4)
*Referred with suspected recurrence of previous sarcoma (n (%))*				
Yes	6 (1.6)	0 (0.0)	7 (6.9)	13 (2.4)
No	369 (98.4)	68 (100.0)	95 (93.1)	532 (97.6)

^1^One patient presented to her father who was a doctor, one patient was a doctor and referred himself, and one patient presented to a friend who was a doctor.

^2^Percentages calculated from the total of patients who had data available for this variable, meaning that the patient had been seen by their GP and the GP had provided an answer for this question (*n* = 307 for benign conditions, *n* = 47 for other malignancies, *n* = 82 for sarcomas, and *n* = 436 for the total population).

^3^Trend towards a higher number for sarcoma patients compared to patients with benign conditions (*p* = 0.051).

^4^Significantly higher for sarcoma patients compared to patients with benign conditions (*p* = 0.001) and for patients with other malignancies compared to patients with benign conditions (*p* < 0.001).

**Table 5:** Median number of days (interquartile intervals) spent in each interval of the diagnostic process from first symptom to decision of diagnosis/treatment.

	Patient interval median (IQI)	Primary care interval median (IQI)	Local hospital interval median (IQI)	Sarcoma centre interval median (IQI)	Diagnostic interval median (IQI)	Total interval median (IQI)
	*n* = 545	*n* = 416	*n* = 386	*n* = 545	*n* = 545	*n* = 545
*All patients*	54 (12 : 241)	8 (1 : 36.5)	26.5 (13 : 58)	15 (9 : 22)	50 (30 : 98)	155 (61 : 423)
*Gender*						
Female	48.5 (9 : 182)	11 (1 : 39.5)	23 (13 : 60)	16 (11 : 23)	52 (31 : 98)	144.5 (60 : 341)
Male	59 (13 : 319)	4 (1 : 35)	28 (13 : 54)	15 (8 : 22)	50 (29 : 99)	158 (62 : 507)
*Age*						
<20	31 (15 : 84)	22 (2 : 73)	21 (11 : 58)	15 (8 : 20)	55 (30 : 139)	118 (47 : 259)
20–39	76 (21 : 539)	12 (1 : 49)	36.5 (18.5 : 102)	17 (11 : 25)	57 (33 : 148)	184 (77 : 924)
40–59	110 (17 : 349)	7.5 (1 : 36)	32.5 (16 : 72)	15 (8 : 22)	62 (31 : 106)	225 (78 : 591)
≥60	36.5 (4 : 134)	3.5 (1 : 33)	21 (11 : 43)	15 (9 : 23)	42.5 (27 : 78)	99 (46 : 240)
*Pt had or developed lump*						
No	38.5 (1 : 215)	22 (4 : 58)	24 (9 : 67)	19 (11 : 28)	57.5 (35 : 116.5)	147 (49.5 : 342.5)
Yes	59 (17 : 251)	3 (1 : 31)	28 (15 : 54)	15 (9 : 21)	49 (28 : 98)	156 (63 : 507)
*Patient had or developed pain*						
No	33.5 (3 : 236.5)	1 (1 : 31)	23.5 (12 : 47)	15 (9 : 21.5)	41 (26 : 84)	95 (43.5 : 389.5)
Yes	76 (20 : 241)	13 (1 : 44)	29 (14 : 65.5)	16 (9 : 22)	58 (34 : 134)	182 (77 : 465)
*Tumour size* ^1^						
Under 5 cm	46 (9 : 200)	12 (1 : 40)	30 (15 : 62)	15 (8 : 22)	56 (32 : 106)	147 (59 : 383)
Over 5 cm	63 (15 : 319)	14 (1 : 45)	23 (13 : 52)	16 (9 : 23)	57 (33 : 107)	180 (76.5 : 571)
*Tumour depth* ^2^						
Subcutaneous	86 (15 : 528)	1 (1 : 36)	28 (15 : 54)	13 (8 : 20)	42 (28 : 91)	181 (60 : 734)
Subfascial	58.5 (14 : 234)	7 (1 : 29)	29 (15 : 56)	15 (9 : 21)	55 (31.5 : 100.5)	147 (65 : 416)
*GP suspected malignancy at initial referral* ^3^						
No	81 (22 : 319)	9 (1 : 45)	38 (20 : 78)	15 (9 : 22)	63 (38 : 139)	197 (90 : 690)
Yes	45 (11 : 141)	4 (1 : 25)	18 (9.5 : 28)	15 (8 : 21)	34 (21 : 58)	94 (45 : 215)
*Referred from Aarhus local uptake area*						
No	55 (11 : 227)	8 (1 : 40)	28 (15 : 58)	15 (9 : 22)	35 (21 : 88)	158 (63 : 401)
Yes	43 (13 : 323)	3 (1 : 28)	18 (6 : 47)	16 (9 : 25)	56 (33 : 106)	135 (51 : 469)
*Diagnosis*						
Sarcomas	77 (11 : 261)	17 (1 : 56)	29 (15 : 56)	17 (10 : 24)	65 (42 : 133)	176 (83 : 673)
Other malignancies	38 (6 : 97)	12.5 (1 : 25)	15 (7 : 32)	20 (14 : 26)	44 (27.5 : 68)	103 (49.5 : 202.5)
Benign	54 (13 : 296)	4 (1 : 35)	28 (16 : 62)	15 (8 : 21)	48 (29 : 91)	158 (59 : 507)
*Malignancy grade* ^4^						
Low-grade	213 (26 : 963)	21.5 (1 : 50)	29 (19 : 47)	17 (8 : 23)	60 (43 : 103)	250 (108 : 1665)
High-grade	41 (8 : 154)	17 (1 : 57)	29 (13 : 58)	17 (13 : 25)	71 (42 : 140)	164 (69 : 376)

*n* = total number of patients with available dates for calculation of this interval.

^1^Analysis included only patients with data for tumor size measured on MRI/CT or histology.

^2^Analysis included only patients with soft tissue tumors.

^3^Analysis included only patients with data available for this variable. Patients who were not seen by the GP and patients where the GP had not answered the question were excluded from the analysis.

^4^Analysis included only sarcoma patients.

**Table 6:** Estimated differences in time intervals at the 50th and 75th percentiles, measured as difference in calendar days with 95% confidence intervals (CI), calculated by quantile regression.

	Patient interval	Primary care interval	Local hospital interval	Sarcoma centre interval	Diagnostic interval	Total interval
	Estimate (95% CI)	Estimate (95% CI)	Estimate (95% CI)	Estimate (95% CI)	Estimate (95% CI)	Estimate (95% CI)
*Sarcoma patients versus patients with benign conditions*						
50th percentile	16 (−37 : 69)	**10** (**4 : 15**)	0 (−9 : 10)	0 (−9 : 10)	**19 **(**10 : 28**)	26 (−34 : 86)
75th percentile	−7 (−18 : 5)	**24** (**9 : 39**)	−2 (−12 : 9)	−2 (−12 : 9)	**30** (**14 : 45**)	**206** (**145 : 267**)
*Patients with other malignancies versus patients with benign conditions*						
50th percentile	−**21** (**−30 : −12**)	9 (−3 : 22)	**−13** (**−18 : −8**)	**5** (**3 : 8**)	−2 (−9 : 6)	**−47** (**−60 : −34**)
75th percentile	**−211** (**−226 : −196**)	**−6** (**−11 : −2**)	−**27** (**−36 : −17**)	**4** (**0 : 7**)	−15 (−43 : 13)	**−285** (**−296 : −274**)
*Patients presenting with a lump versus patients presenting without a lump*						
50th percentile	26 (−3 : 56)	**−19 **(**−26 : −12**)	4 (−2 : 10)	**−4 **(**−7 : −1**)	−9 (−22 : 3)	−4 (−40 : 31)
75th percentile	**39** (**11 : 67**)	**−30** (**−42 : −17**)	**−21** (**−28 : −14**)	**−7 **(**−10 : −3**)	−11 (−52 : 29)	**121** (**75 : 167**)
*Patients presenting with pain versus patients presenting without pain*						
50th percentile	**40** (**18 : 61**)	**12** (**1 : 23**)	5 (−3 : 13)	1 (0 : 3)	**17** (**12 : 21**)	**78 **(**60 : 96**)
75th percentile	19 (−10 : 47)	**14** (**5 : 23**)	**16** (**7 : 26**)	1 (−2 : 4)	**37 **(**23 : 51**)	**82** (**58 : 105**)
*Patients where GP initially suspected malignancy versus patients where GP did not suspect malignancy* ^1^						
50th percentile	**−41** (**−54 : −28**)	−1 (−12 : 10)	**−20** (**−29 : −11**)	−1 (−2 : 1)	−31 (−68 : 7)	**−104** (**−117 : −91**)
75th percentile	**−187** (**−202 : −171**)	**−21 **(**−28 : −15**)	**−50** (**−62 : −38**)	−2 (−5 : 2)	−**74** (**−112 : −35**)	**−480** (**−516 : −445**)
*Tumour size over 5 cm versus tumour size under 5 cm* ^2^						
50th percentile	**24** (**2 : 46**)	5 (−21 : 31)	−6 (−14 : 3)	1 (−1 : 4)	−1 (−12 : 10)	**43** (**27 : 59**)
75th percentile	**170** (**131 : 210**)	6 (−6 : 18)	−7 (−21 : 8)	2 (−3 : 7)	9 (−9 : 28)	**225** (**174 : 275**)
*Subfascial depth versus subcutaneous depth* ^3^						
50th percentile	**−31** (**−49 : −12**)	2 (−2 : 5)	3 (−8 : 14)	1 (−1 : 3)	**9 **(**2 : 16**)	−34 (−81 : 13)
75th percentile	**−306** (**−319 : −293**)	−2 (−17 : 13)	−4 (−16 : 7)	2 (0 : 4)	5 (−9 : 19)	**−296** (**−309 : −283**)
*High-grade tumours versus low-grade tumours* ^4^						
50th percentile	**−160** (**−191 : −129**)	−1 (−11 : 8)	0 (−5 : 5)	0 (−4 : 4)	**21** (**11 : 31**)	**−104** (**−110 : −98**)
75th percentile	−**1195** (**−1281 : −1110**)	7 (−2 : 16)	**20** (**11 : 28**)	**4** (**1 : 7**)	**38** (**29 : 46**)	**−1270** (**−1288 : −1253**)

All estimates are adjusted for age. Bold numbers indicate statistical significance at the 5% level.

^1^Analysis included only patients with soft tissue tumors.

^2^Analysis included only patients with data for tumor size measured on MRI/CT or histology.

^3^Analysis included only patients with data available for this variable. Patients who were not seen by the GP and patients where the GP had not answered the question were excluded from the analysis.

^4^Analysis included only sarcoma patients.

## Appendix

For more details see Tables 2 and 3.
